# Multiple white flat lesions on upper endoscopy: a systematic review and meta-analysis of the association with proton pump inhibitor exposure

**DOI:** 10.1186/s12876-026-04771-z

**Published:** 2026-05-07

**Authors:** Liang Zhang, Jian Liu, Yali Wang

**Affiliations:** Department of Gastroenterology, The First Hospital of Tsinghua University, No. 6, Jiuxianqiao 1st Neighborhood, Chaoyang District, Beijing, China

**Keywords:** Helicobacter pylori, Haruma–Kawaguchi lesions, Multiple white flat lesions, Proton pump inhibitors, Image-enhanced endoscopy, Biopsy, Systematic review

## Abstract

**Background:**

Recognition of multiple white flat lesions (MWFLs), or white flat elevated mucosa (WFEM), is increasing. Their tendency to mimic gastric intestinal metaplasia often leads to unwarranted biopsies. To address this, we reviewed existing evidence covering their endoscopic characteristics, relationship with H. pylori, detection frequencies, and longitudinal course, including the observational association with proton pump inhibitor (PPI) therapy.

**Methods:**

From 1 January 2010 to 30 November 2025, we searched PubMed, Embase, Web of Science, Scopus, CENTRAL, and East Asian databases without language restrictions. We screened the reference lists of included studies. We pooled study-reported multivariable-adjusted odds ratios (ORs) for PPI exposure using a random-effects model wherever possible. Detection frequencies were reported descriptively, as pooling was not appropriate given the ascertainment heterogeneity.

**Results:**

We retained five observational studies (*N* = 5,065). Pooling was feasible for three studies contributing cross-sectional exposure-outcome estimates (k = 3; Japan *n* = 2, China *n* = 1; *N* = 2,907) reporting multivariable-adjusted ORs. In this limited exploratory synthesis, PPI exposure, however defined, was associated with higher odds of MWFLs (pooled adjusted OR 2.91, 95% CI 1.98–4.29; I²=16.9%). As all studies were at moderate risk of bias, residual confounding and detection bias cannot be excluded. Study-level detection frequencies in four unselected cohorts (*N* = 4,902) ranged from 3.0% to 10.4% (these are detection frequencies, not population prevalence estimates). Regardless of how variables were defined, MWFLs were typically associated with *H. pylori*-negative or post-eradication status rather than active infection. Longitudinal evidence was inadequate to determine malignant potential or whether biopsy can be safely deferred.

**Conclusions:**

Uncertainty regarding the malignant potential of typical MWFLs precludes any conclusion on the safety of biopsy omission. In the limited available cross-sectional evidence, PPI exposure was associated with higher MWFL odds, but this estimate should be viewed as hypothesis-generating rather than causal. Careful description of suspected MWFLs, best achieved with image-enhanced endoscopy, is essential while prospective, protocol-driven investigations resolve questions regarding their natural history, malignant potential, and the resolution of diagnostic uncertainty.

**Registration:**

PROSPERO CRD420251248816.

**Supplementary Information:**

The online version contains supplementary material available at 10.1186/s12876-026-04771-z.

## Background

Gastric cancer remains a major global health burden, particularly in East Asia, with substantial incidence and mortality reported worldwide [1, 2]. Multiple white flat lesions (MWFLs)—variously termed white flat elevated mucosa (WFEM) or Haruma–Kawaguchi lesions—are a frequent endoscopic finding. Their visual resemblance to gastric intestinal metaplasia (IM) poses a diagnostic challenge, however, often prompting unnecessary biopsies and patient anxiety [3, 4]. While early Japanese reports characterized these as multiple white, flat, or slightly elevated lesions in the gastric body or fundus [3, 4], subsequent data implicated acid-suppressive therapy, specifically proton pump inhibitors (PPIs) [5–9]. Histologically, these lesions manifest as non-neoplastic oxyntic mucosa with foveolar hyperplasia and minimal inflammation, without goblet cells. Regular microsurface and microvascular patterns are typically discernible via image-enhanced and magnifying endoscopy [3, 5, 10]. Having been incorporated into the Kyoto classification of gastritis [11–14], MWFLs aid in gastric-cancer risk stratification [15–18]. Rare during active H. pylori infection, they predominate in H. pylori-negative or post-eradication stomachs, particularly among long-term PPI users [5–9]. Heterogeneity in case definitions, endoscopic techniques, and exposure criteria likely underpins the broad variation in reported detection frequencies [5–9].

The existing literature on MWFLs lacks granularity, particularly due to the scarcity of quantitative PPI data and the wide divergence in detection frequencies observed across varying contexts. Direct evidence on malignant potential or biopsy strategy is also limited. We therefore conducted a systematic review with a limited, hypothesis-generating meta-analysis to synthesize the available literature on MWFLs. Our specific objectives were to: (1) characterize reported endoscopic and clinicopathologic features, H. pylori context, and longitudinal outcomes; (2) report study-level MWFL detection frequencies in unselected endoscopy cohorts; and (3) provide a limited quantitative summary of the association between PPI exposure and MWFLs, provided sufficiently comparable data existed; research questions and outcomes were prespecified according to the PECOS/PICO framework.

## Methods

### Protocol and registration

The review methods were pre-specified in the protocol registered with PROSPERO (CRD420251248816). The review is reported in accordance with the PRISMA 2020 statement; the checklist is provided with this submission. The full protocol can be accessed via the PROSPERO record (CRD420251248816). AI-based language tools (including large language models) were used to assist with manuscript drafting and editing; all content was reviewed and verified by the authors.

### Eligibility criteria

We included studies of adults undergoing upper gastrointestinal endoscopy that defined MWFLs (or white flat elevated mucosa/synonyms) as an endoscopic observation and that yielded extractable data for detection frequency, exposure association, H. pylori status, or longitudinal outcomes. We excluded studies that did not clearly distinguish MWFLs from other gastric lesions, as well as case reports/series without a denominator and conference abstracts without sufficient data.

Our eligibility criteria, based on the PECOS framework, were as follows: Participants: adults receiving an upper endoscopy; Exposure: proton pump inhibitor use (current or chronic, according to study-specific definitions). Non-PPI users served as controls, and the outcome was multiple white flat lesions (MWFLs). The accepted study designs were observational (cross-sectional or prospective).

### Information sources and search strategy

PubMed, Embase, Web of Science, Scopus, CENTRAL, CiNii, J-STAGE, CNKI, Wanfang, VIP, KoreaMed, and RISS were searched up to November 30, 2025, without language restrictions. Owing to the descriptive orientation of earlier reports—and their lack of extractable denominators for statistical pooling—we limited inclusion to studies published from January 1, 2010, onward. The full electronic search strategies are provided in Additional file 1. Supplementary Tables S1–S8 are provided in Additional file 2, Supplementary Figures S1–S6 in Additional file 3, and the PRISMA 2020 checklist in Additional file 4. Cross-referencing the bibliographies of included articles and relevant reviews yielded no further eligible studies. No searches were performed after 30 November 2025, and we did not conduct additional gray literature searches.

### Study selection

Two reviewers independently screened titles and abstracts once duplicates were removed. Full-text reports were then evaluated for eligibility. Any differences were resolved through discussion; if we could not agree, a third reviewer made the final decision.

### Data extraction and quality assessment

Using a standardized extraction template, L.Z. and J.L. independently compiled the dataset; discrepancies were resolved by consensus. We extracted data on MWFL definitions, endoscopic modalities (white-light or image-enhanced), and *H. pylori* status. We also recorded PPI exposure metrics—drug, dose, and duration—and documented the variable definitions of ‘current’ and ‘long-term’ use across studies. Adjusted ORs, 95% CIs, exposure definitions, and covariates were collated and assigned to the respective quantitative syntheses (Table S6 and Table S6 in Additional file 2). Overall summaries included those with unknown *H. pylori* status; however, these individuals were omitted from stratified analyses unless robust reclassification was possible. Study-specific categories guided stratification. Because the original studies classified never-infected and post-eradication participants inconsistently, we combined them into a single “*H. pylori*-negative/post-eradication” category while acknowledging the underlying clinical heterogeneity. We also captured details on *H. pylori* ascertainment methods (test modality; handling of untested/unknown cases) where reported.

Two authors (L. Z. and J. L.) assessed the risk of bias of included studies using the ROBINS-I tool. Disagreements were resolved by consensus. A summary of risk of bias is presented in the Results section and detailed in Table S4. NOS scores were also calculated for comparison (Table S3/Figure S3).

### Statistical analysis

All statistical analyses were performed in Stata 19.5 (StataCorp LLC, College Station, TX, USA), using the Stata meta-suite. Because the quantitative data were sparse and clinically heterogeneous, we used narrative synthesis to summarize lesion characteristics, *H. pylori* context, and longitudinal outcomes. The unselected endoscopy cohort was defined as screening or consecutive routine endoscopy populations that were not enriched for MWFLs or PPI exposure. Our limited quantitative synthesis consisted of a random-effects meta-analysis of multivariable-adjusted odds ratios (ORs) from studies contributing cross-sectional exposure-outcome estimates, contrasting PPI users against non-users; given the small number of studies, this pooled estimate was considered exploratory in nature. Because operational definitions of PPI exposure varied across studies (Table 1; Table S6), the pooled estimate represents an average effect across these disparate definitions. We pooled log-transformed ORs and back-transformed them to obtain summary estimates with 95% confidence intervals (CIs).

**Table 1:** Characteristics of included studies

Study	Country	Design/Setting	Population (*N*)	PPI exposure definition	Key findings
Adachi et al. 2018 [6]	Japan	Cross-sectional; single-center screening endoscopy (unselected cohort)	Health check-up EGD; *N* = 1,995	Current PPI use (very few users)	MWFLs were reported more often after *H. pylori* eradication; inference about the PPI association was limited by the very small number of users.
Hatano et al. 2018 [8]	Japan	Cross-sectional; multicenter routine diagnostic endoscopy (unselected cohort)	Outpatients undergoing EGD; *N* = 1,214	Current PPI use vs. non-use	Current PPI use and prior *H. pylori* eradication therapy were associated with higher MWFL detection (termed white flat elevated mucosa in that study).
Majima et al. 2018 [7]	Japan	Prospective study; single-center screening endoscopy (unselected cohort) with follow-up subset	Screening EGD; *N* = 767	Long-term PPI use (≥ 6 months)	Long-term PPI use was associated with higher odds of MWFLs; in the limited follow-up subset, most lesions appeared stable and no malignant transformation was reported.
Hasegawa et al. 2024 [5]	Japan	Prospective observational; single-center (enriched *H. pylori*-negative cohort)	*H. pylori*-negative: 71 long-term PPI users vs. 92 non-users (*N* = 163)	Oral PPI intake (long-term)	MWFL frequency was approximately 4.5-fold higher among PPI users; oral PPI intake remained independently associated with MWFLs in this enriched cohort; not population-representative.
Zhou et al. 2020 [9]	China	Cross-sectional; single-center diagnostic endoscopy (unselected cohort)	Consecutive diagnostic EGD; *N* = 926	Current PPI use vs. non-use	MWFLs were reported more often among PPI users; pathology was described as hyperplastic polyp-like changes (Chinese-language report).

We extracted data for Zhou et al. [9] from the original Chinese text. Majima et al. [7] was reported as a prospective study, but its baseline exposure-outcome estimate was eligible for the primary synthesis because it reflected a single-time-point association. The follow-up subset is summarized separately

*Abbreviations*: *MWFLs* Multiple white flat lesions, *EGD* Esophagogastroduodenoscopy, *PPI* Proton pump inhibitor, *H. pylori* Helicobacter pylori, *OR* Odds ratio

Restricted maximum likelihood (REML) was used to estimate between-study variance (τ²). To obtain more conservative inference when the number of studies is small, we applied the Hartung–Knapp adjustment as a sensitivity analysis. Prespecified sensitivity analyses included the addition of a single prospective observational study and the combination of crude ORs from extracted 2 × 2 tables, applying a 0.5 continuity correction to cells with zero entries. Heterogeneity assessment employed I² and τ² measures. We planned to assess small-study effects using funnel plots and Egger’s test when ≥ 10 studies were available. Because fewer than 10 studies were available for any synthesis, we did not perform Egger’s test. The funnel plot (Supplementary Figure S5) for the crude OR sensitivity analysis is descriptive and cannot be used to infer publication bias.

### Protocol amendments (deviations from protocol)

While our original study protocol included a pooled prevalence analysis, we ultimately re-evaluated the feasibility of meta-analyzing MWFL detection frequencies. Because the ascertainment of MWFLs is so heavily influenced by the diagnostic setting and the awareness of the endoscopist, we found that a combined prevalence figure would lack the required interpretability. We provide a descriptive summary of detection frequencies and restrict our quantitative meta-analysis to the link between PPI exposure and MWFLs.

### Certainty of evidence

Assessments of evidence certainty for the primary outcomes are reported in the Results, adhering to the GRADE framework. These assessments are based on the risk of bias, inconsistency, indirectness, imprecision, and publication bias.

## Results

### Study selection

**Fig. 1:**
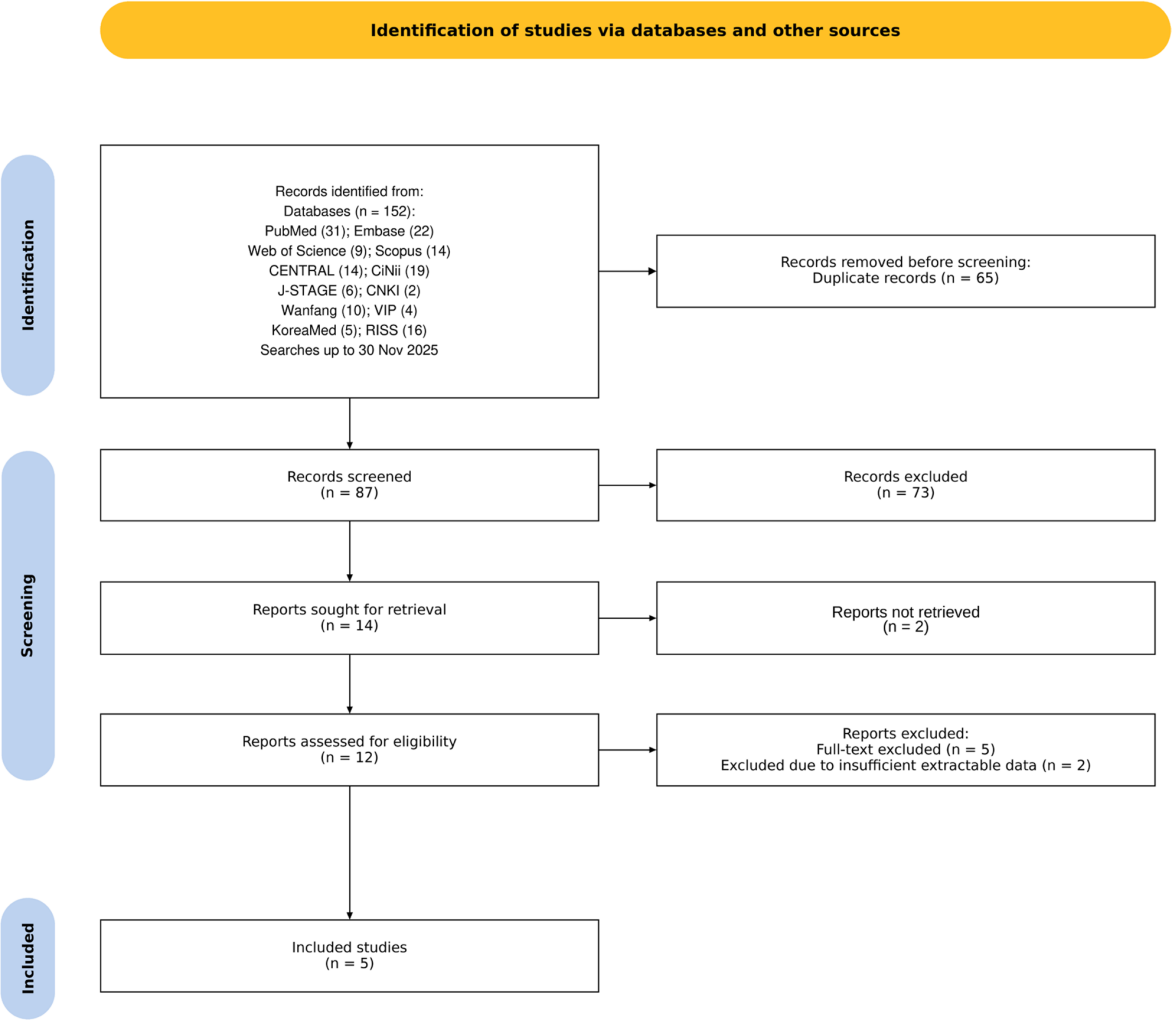
PRISMA 2020 flow diagram. Numbers indicate records at each stage of screening and eligibility assessment

The process of study selection, following the PRISMA 2020 framework, is depicted in Fig. 1. Five observational studies were eligible for inclusion. Table S2 lists the specific reasons for full-text exclusions. We excluded Hasegawa et al. [19] and Zhou & Duan [20] owing to missing denominators or PPI-specific effect estimates required for analysis. To prevent duplication from overlapping cohorts, the largest dataset was consistently selected. Two reports could not be obtained.

### Study characteristics

Five observational studies published between 2018 and 2024 were included. Cross-sectional designs accounted for three studies. Additionally, we included one prospective study (using its baseline exposure-outcome estimate for the primary synthesis) and one prospective observational study of an enriched H. pylori-negative cohort. Four studies were conducted in Japan and one in China, with a total of 5,065 adults undergoing upper gastrointestinal endoscopy [5–9]. The operative definitions of MWFLs were largely consistent across studies (defined as multiple white, flat/slightly elevated lesions on the gastric mucosa), although there were differences in the operational definition of PPI exposure (e.g., current vs. chronic use and varying duration thresholds), the ascertainment and categorization of H. pylori status (e.g., positive/negative/unknown and differences in handling untested individuals), and endoscopic imaging techniques (Table 1). Definitional divergences likely account for the wide variation in reported detection rates, limiting the inference possible from pooled frequency data. Specifically, the distinction between “current” and “long-term” PPI exposure—along with the duration thresholds applied—varied across the dataset (Table 1). The aggregate estimate thus constitutes an average effect spanning these disparate definitions.

### Risk of bias

**Fig. 2:**
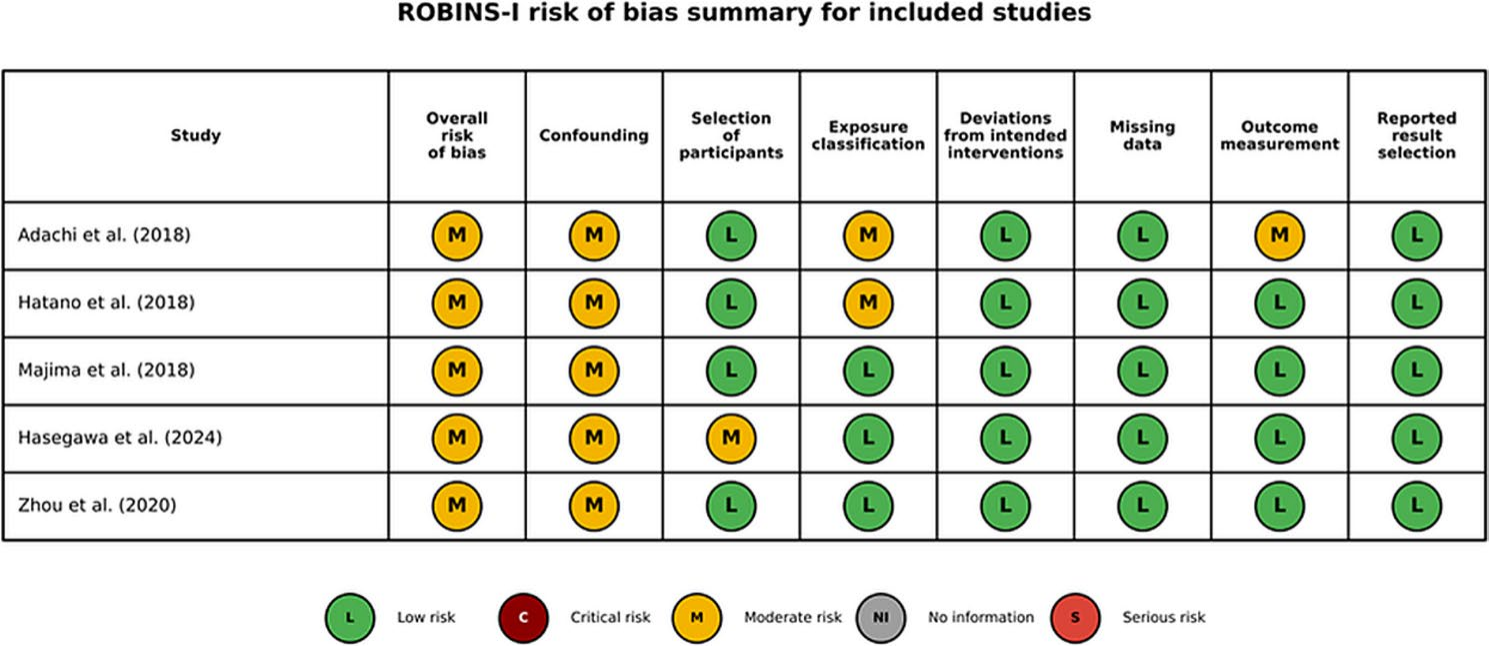
ROBINS-I risk-of-bias summary for included studies (k = 5; Japan *n* = 4, China *n* = 1). Shown here are study-level judgments across all seven domains and the overall risk. Refer to Table S4 for domain-by-domain ratings and key concerns

We graded the overall risk of bias as moderate across all studies (Fig. 2; Table S4). We attributed this classification primarily to residual confounding (e.g., PPI indication, *Helicobacter pylori* infection) and exposure classification. Detection bias was also considered, because endoscopists were typically not blinded to the patients’ medication history.

### Limited quantitative summary of the reported association between PPI exposure and MWFLs

Five observational studies qualified for synthesis. Owing to the thin and heterogeneous evidence base, we adopted a narrative synthesis for lesion characteristics, H. pylori associations, and longitudinal outcomes. Data were pooled quantitatively only if the multivariable-adjusted odds ratios from included studies were appropriate for meta-analysis.

**Fig. 3:**
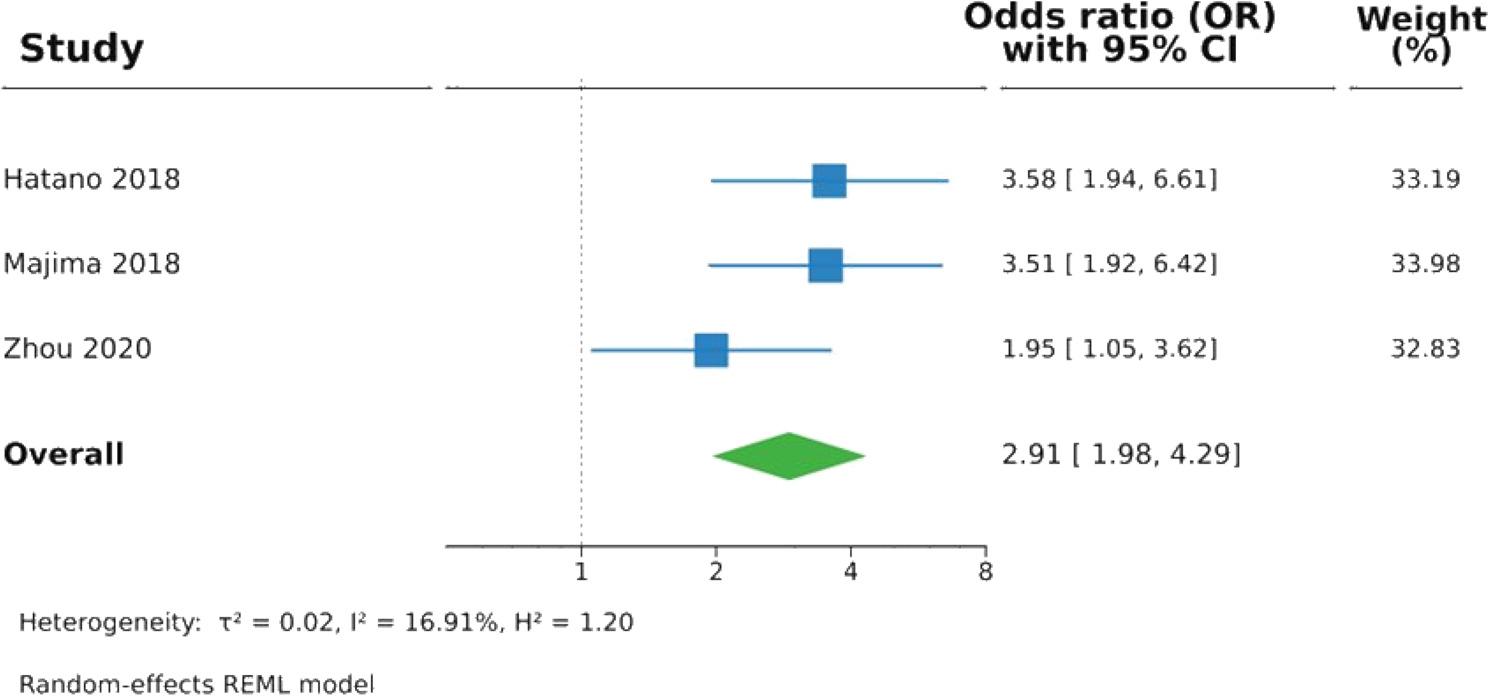
Adjusted association between PPI exposure and MWFLs. We sourced cross-sectional exposure-outcome estimates from three studies (Japan *n* = 2; China *n* = 1; *N* = 2,907). A random-effects REML model was used to pool the three study-reported multivariable-adjusted odds ratios. Study-specific estimates (95% CIs) appear as squares, while the diamond signifies the pooled estimate. As these estimates were observational and at moderate risk of bias, the pooled result is intended to generate hypotheses rather than confirm them and should not be interpreted as causal. Figure S4 in Additional file 3 shows the sensitivity analysis including one prospective observational study (k = 4)

Multivariable-adjusted ORs from three cross-sectional studies (*N* = 2,907; Japan, *n* = 2; China, *n* = 1) were deemed sufficiently comparable for meta-analysis. PPI exposure, as defined by the respective authors (Table 1 and Table S6), was associated with an increased likelihood of MWFLs (pooled adjusted OR 2.91, 95% CI 1.98–4.29; I²=16.9%; Fig. 3). Given the cross-sectional nature of the data, the observed association is synchronous, precluding inferences regarding temporal sequence. Given that the observational evidence was characterized by a moderate risk of bias and absent endoscopist blinding, these results remain vulnerable to detection bias and residual confounding—specifically confounding by indication. Therefore, a causal relationship cannot be established from these data. The pooled adjusted OR stayed > 1 (3.54, 95% CI 2.31–5.45; I²=0.0%; k = 2; *N* = 1,981) even after excluding Zhou et al. [9] for its lack of H. pylori adjustment.

**Fig. 4:**
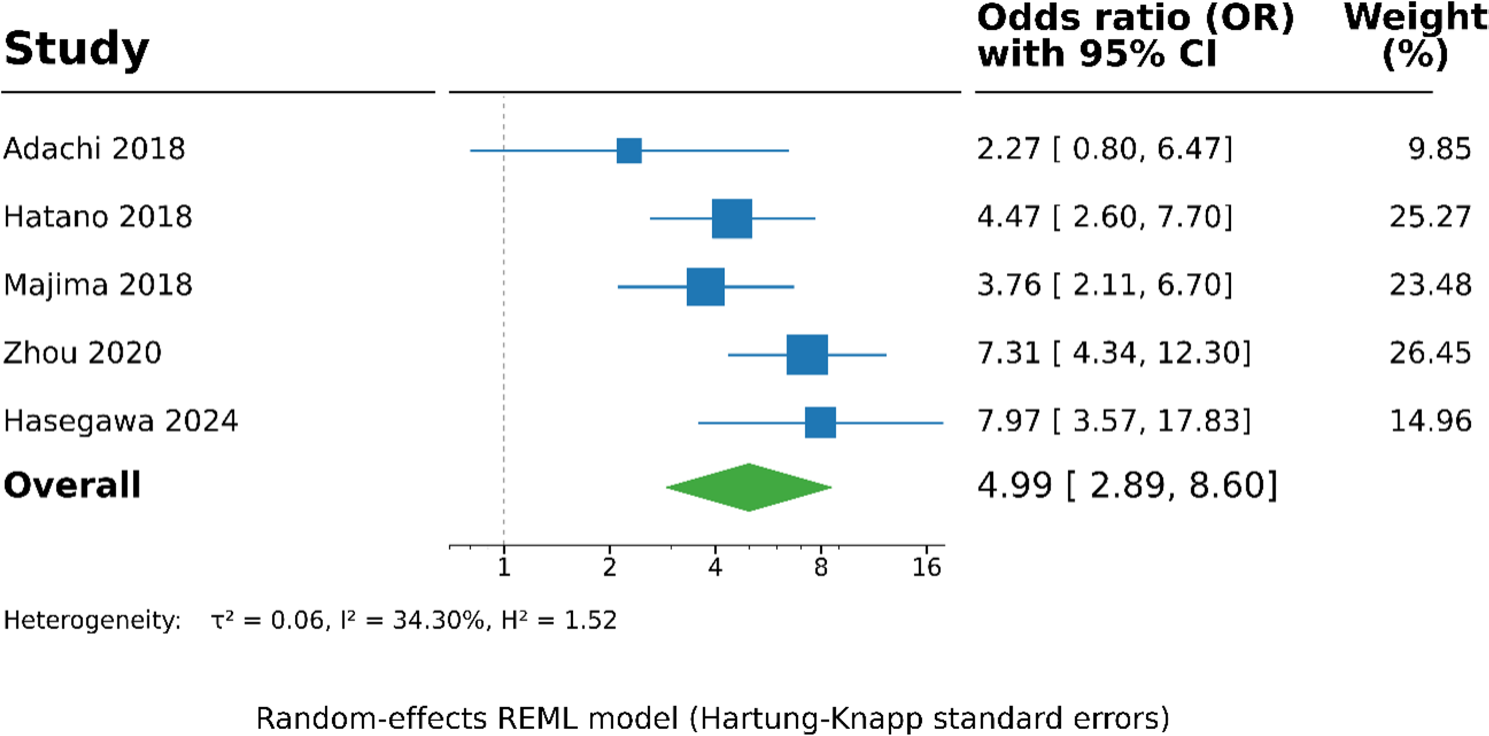
Crude OR sensitivity analysis. Five observational studies were included (Japan *n* = 4, China *n* = 1; *N* = 5,065). Crude ORs were generated from unadjusted 2 × 2 contingency tables for PPI exposure vs. non-exposure. In this figure, the squares correspond to study-specific ORs (95% CIs), whereas the diamond represents the pooled result from a random-effects REML model using the Hartung–Knapp modification. Note that this analysis is purely descriptive and lacks adjustment for confounding by indication. A constant of 0.5 was added for zero-cell correction before computing the ORs

Because all contributing studies were rated at moderate (instead of serious/high) risk of confounding bias, filtering for low-to-moderate risk had no impact on the selection of studies. Use of the Hartung–Knapp adjustment widened the CI (adjusted OR 2.91, 95% CI 1.24–6.84; k = 3). A directionally similar estimate emerged when one prospective observational study was added (k = 4; *N* = 3,070; pooled adjusted OR 3.17, 95% CI 2.17–4.62; I²=19.9%) (Figure S4 in Additional file 3), but this does not overcome the fundamental limitations of observational, geographically limited data. Table 2 presents the results of the primary and sensitivity analyses; the descriptive sensitivity analysis for crude OR is depicted in Fig. 4. Supplementary Table S8 in Additional file 2 provides descriptive unadjusted MWFL proportions.

**Table 2:** Sensitivity analyses for primary adjusted OR meta-analysis (PPI vs. no PPI)

Analysis scenario	Pooled OR (95% CI) for PPI vs. no PPI
Primary (cross-sectional estimates only; k = 3; Japan *n* = 2, China *n* = 1; *N* = 2,907)	2.91 (1.98–4.29)
Sensitivity (Hartung–Knapp adjustment; cross-sectional estimates only; k = 3; Japan *n* = 2, China *n* = 1; *N* = 2,907)	2.91 (1.24–6.84)
Sensitivity (including one prospective observational study; k = 4; Japan *n* = 3, China *n* = 1; *N* = 3,070)	3.17 (2.17–4.62)
Post-hoc confounding sensitivity (excluding Zhou 2020; least-adjusted model; k = 2; Japan only; *N* = 1,981)	3.54 (2.31–5.45)

### Secondary outcome: incidence of MWFLs detection across unselected study-reported endoscopy cohorts

Detection of MWFLs among 4,902 unselected endoscopy patients from four screening/routine cohorts ranged from 3.0% to 10.4% (Table 1). Such inconsistencies are presumably attributable to varying cohort profiles, imaging techniques, and the level of endoscopist expertise. It is important to note that these figures are cohort-dependent rather than representative of the population at large.

### Secondary outcomes and clinical course

#### Other factors

The available literature indicates that associations between MWFLs and demographic traits (age, sex) are inconsistent once drug exposure has been accounted for [7–9].

#### *H. pylori* status

MWFLs were more common in *H. pylori*-negative participants (never infected or post-eradication) than in *H. pylori*-positive participants (pooled unadjusted OR = 1.86; 95% CI, 1.15–3.00) (Figure S2). No statistical heterogeneity was found (I² = 0.0%). However, this result is based on only two studies (k = 2), making I² an imprecise estimate. This analysis is exploratory. The *H. pylori*-negative group was not uniform; it included both never-infected and post-eradication individuals. Definitions and testing protocols for *H. pylori* were not uniform, leading to the inclusion of untested groups in some studies. These were kept in the overall results but excluded from stratified analyses unless reclassification was achievable, as described in the Methods.

### Clinical course

Longitudinal data are scant. In the single cohort detailing follow-up (mean 2.3 years), lesions appeared endoscopically stable without malignant transformation; yet, the selection and size of this subset were not explicitly defined [7]. Although therapy discontinuation led to regression in two instances, denominators were not reported [7]. This paucity of rigorous follow-up data precludes confident exclusion of rare dysplasia or malignancy and prevents any determination of whether biopsy may be safely deferred for typical lesions. Regression of other PPI-related pathologies (e.g., fundic gland polyps) is documented in case reports [21].

### Certainty of evidence

According to GRADE, certainty was rated very low for MWFL detection frequencies because of serious indirectness (setting-specific ascertainment), inconsistency, and imprecision. Certainty for the association between PPI exposure and MWFLs was rated low because the primary synthesis is based on only three studies contributing cross-sectional estimates from two countries, and all studies were at moderate risk of bias, with likely residual confounding and detection bias (Table 3). Even with directionally comparable estimates from sensitivity analyses (omitting the least adjusted study; including a prospective cohort), these limitations are not overcome. Hence, we treat the pooled estimate as preliminary, distinct from a definitive result.

**Table 3:** Summary of findings and certainty of evidence (GRADE-based)

Outcome	Effect estimate	Studies (*N*)	GRADE	Notes
Association: PPI use vs. no PPI	Adjusted OR 2.91 (95% CI: 1.98–4.29); I²=16.9%.	3 (*N* = 2,907)	Low	Certainty of evidence was lowered because of risk of bias (consistently moderate (domain-level judgment) per ROBINS-I), indirectness (exclusively East Asian data), and imprecision (small k = 3, cross-sectional estimates). Accordingly, we regard the pooled estimate essentially as hypothesis-generating in nature. Because no studies reached a serious or high risk of confounding bias, filtering for low/moderate risk left the eligible studies unchanged. Estimates remained directionally similar in sensitivity analyses that excluded Zhou (2020) and included a prospective cohort, although the inherent weaknesses of observational evidence are not eliminated.
MWFLs detection frequency in unselected endoscopy cohorts (descriptive)	3.0%–10.4% across cohorts (Table S1).	4 (*N* = 4,902)	Very low	Case ascertainment variables (study setting, cohort mix, endoscopy modality, and operator awareness) substantially affect frequency estimates. Thus, these figures reflect setting-specific detection frequencies rather than a single generalizable prevalence.
Association: *H. pylori*-negative vs. *H. pylori*-positive	Exploratory unadjusted OR 1.86 (95% CI: 1.15–3.00); I²=0.0%.	2 (*N* = 1,981)	Low	Risk of bias and imprecision; *H. pylori*-negative combined never-infected and post-eradication categories.
Malignant transformation during follow-up	0 malignant transformations reported in the only cohort with follow-up (mean 2.3 years).	1 (follow-up subset; n not reported)	Very low	Very sparse longitudinal data; follow-up subset size not reported; imprecision and risk of bias.

## Discussion

The most pressing clinical questions concerning MWFLs involve their potential for malignancy and whether biopsy can be safely deferred in classic cases. Direct evidence addressing these issues is currently sparse. Consequently, this review synthesized a limited, geographically concentrated dataset; we also pooled available adjusted estimates of the PPI-MWFL association as an exploratory secondary measure. Only three cross-sectional studies from two countries contributed adjusted estimates to the primary synthesis. Consequently, the pooled adjusted OR of 2.91 warrants interpretation as exploratory rather than confirmatory, given the high probability of residual confounding (e.g., by indication) and detection bias. In unselected cohorts, detection rates spanned 3.0% to 10.4%, a variation more likely attributable to differences in setting, technique, and endoscopist awareness than to true population variation. The paucity of longitudinal data leaves the malignant potential of typical MWFLs and whether biopsy deferral is appropriate undetermined.

### Recognizing MWFLs and distinguishing them from intestinal metaplasia

Endoscopically, MWFLs appear as multiple, sharply demarcated whitish lesions in the gastric body or fundus [3–5, 10]. Under magnification or image-enhanced endoscopy, regular microsurface and microvascular patterns are typically seen [3, 5, 10]. Figure S6(Additional file 3) depicts de-identified examples from the fundus and body, visualized via white-light and narrow-band imaging. These images serve purely for visual context and were excluded from review data extraction. Histologically, the lesions manifest as non-neoplastic oxyntic mucosa with foveolar hyperplasia and minimal inflammation, without goblet cells [3, 5, 10]. Nevertheless, given the extreme paucity of longitudinal data—and the fact that one follow-up subset did not report its size [7]—current evidence cannot confidently exclude rare instances of dysplasia or malignant transformation.

### Sources of variation in reported detection frequencies

We observed wide divergence in reported detection frequencies across cohorts. This likely reflects inconsistencies in case definitions, PPI exposure definitions (current vs. long-term; varying duration thresholds), background H. pylori status, endoscopic modality (white-light or image-enhanced), and endoscopist familiarity. Given this degree of clinical and ascertainment heterogeneity, added to the focus on East Asian populations, the interpretability and external validity of a pooled frequency estimate are compromised.

### Clinical implications and reporting considerations

For suspected MWFLs, long-term acid-suppression exposure should be documented whenever available (drug, dose, and duration). However, our review does not determine whether typical MWFLs require biopsy, whether biopsy can be safely deferred, or whether they carry malignant potential. As such, Table 4 offers expert perspectives on reporting standards and conveying persistent diagnostic uncertainty in a pragmatic manner.We offer these as a basis for standardized description and future research, rather than as a tool to direct clinical decision-making.

**Table 4:** Pragmatic (expert-opinion) reporting considerations when MWFLs are suspected

Step	Pragmatic considerations
1. Recognize typical MWFLs	Multiple, sharply demarcated, white, flat/slightly elevated lesions (often gastric body/fundus) with regular microsurface and microvascular patterns.
2. Document gastric background	Record PPI exposure (drug, dose, duration) and background stomach (*H. pylori* status; atrophy/IM).
3. Use IEE when available	Capture representative images under white-light imaging and IEE (e.g., NBI); document distribution (clusters) and borders; archive in the report.
4. Document diagnostic uncertainty	If atypical morphology, irregular patterns, ulceration, focal redness, or IM, dysplasia, or other pathology cannot be confidently excluded, state this uncertainty explicitly in the report; tissue sampling, if undertaken, should follow local standard practice.
5. Record the PPI exposure context	Document the indication for long-term PPI therapy and exposure context (dose and duration) when available. Whether biopsy can be safely deferred in unequivocal cases remains uncertain and requires prospective validation.

These points reflect expert opinion derived from a limited body of observational evidence; they are provided to facilitate standardized reporting and communication of diagnostic uncertainty rather than to function as an evidence-based clinical guideline

Synthesizing current evidence, we propose that extended PPI therapy likely fuels the trophic and morphologic alterations that define MWFLs. The pathogenic pathway is presumably driven by increased gastric pH and the secondary surge in gastrin levels (hypergastrinemia), which together encourage glandular remodeling via epithelial thickening and cystic dilatation [10, 22]. Readers should note, however, that we offer this model as a heuristic tool to orient future study, not as absolute proof of causality. At present, these mechanisms remain conjectural and should be treated as investigational hypotheses.

Clinically, MWFLs are generally more distinct in H. pylori-negative or post-eradication stomachs, whereas active infection tends to obscure their presence [6–8]. Variations in gastric background likely dictate lesion visibility; specifically, inflammatory hyperemia during active H. pylori infection may diminish contrast. This masking phenomenon may partially account for the lower detection rates reported in active infection compared to never-infected or post-eradication cohorts [6–8, 23–26]. Given that PPI-associated lesions appear predominantly in non-infected individuals [5, 7, 8, 27], H. pylori-negative status should not be treated as a monolithic category. The mucosal environments differ substantially, and where data permit, these groups ought to be reported separately. Protocolized prospective cohorts with adjudicated histology are now essential to generate clinically relevant safety data [28–33] and to inform subsequent systematic reviews [34].

### Strengths and limitations

Strengths of this investigation include the adherence to a registered protocol and a comprehensive search strategy spanning Western and East Asian registries. Constraints are imposed by the scarcity and geographic isolation of the data, derived from five observational studies in Japan and China.Significantly, since the primary meta-analysis was based on only three studies with cross-sectional estimates, the pooled finding is best viewed as hypothesis-generating and not causal.Confounding by indication, residual factors (e.g., H. pylori, background gastritis), exposure misclassification, and detection bias from unblinded endoscopy may have influenced the results.Further, the setting-specific variability of these detection frequencies—subject to technical variation—limits their extension to Western clinical practice. The number of studies was insufficient for a reliable assessment of publication bias. Although independent dual screening was conducted, the risk of missing non-indexed or unpublished studies persists.

## Conclusions

To date, studies have not established the malignant potential of typical MWFLs or the safety of omitting biopsy. The available data stem entirely from a handful of observational studies conducted exclusively in East Asia. In a restricted meta-analysis of three cross-sectional studies, PPI usage correlated with higher odds of MWFLs; however, this estimate serves only to generate hypotheses, as methodological biases make it too early to infer causality. Moreover, wide variations in detection frequencies among unselected cohorts rule out their use as reliable indicators of population prevalence. Pending prospective, protocol-driven evidence, suspected MWFLs should be described in detail, preferably via image-enhanced endoscopy, as further inquiry clarifies their natural history, malignant potential, and the appropriate management of diagnostic ambiguity.

## Supplementary Information

Additional file 1. Supplementary Appendix S1. Full electronic search strategies (last search: November 30, 2025).

Additional file 2. Supplementary Tables S1-S8. Supplementary tables (frequencies, exclusions, NOS, ROBINS-I, covariates, descriptive unadjusted proportions).

Additional file 3. Supplementary Figures S1–S6. Supplementary figures (frequency plot, forest plots, quality and risk-of-bias summaries, funnel plot, illustrative endoscopic images).

Additional file 4. PRISMA 2020 checklist.

## Data Availability

All data generated or analyzed during this study are included in this published article and its supplementary information files. The extracted dataset and Stata do-files used for the analyses are available from the corresponding author on reasonable request.

## Abbreviations

CI: Confidence interval
EGD: Esophagogastroduodenoscopy
H. pylori: Helicobacter pylori
IEE: Image-enhanced endoscopy
I²: I-squared
MWFLs: Multiple white flat lesions
NBI: Narrow-band imaging
NOS: Newcastle–Ottawa Scale
OR: Odds ratio
P-CAB: Potassium-competitive acid blocker
PPI: Proton pump inhibitor
PRISMA: Preferred Reporting Items for Systematic Reviews and Meta-Analyses
REML: Restricted maximum likelihood
RoB: Risk of bias
ROBINS-I: Risk Of Bias In Non-randomized Studies of Interventions
WLI: White-light imaging
