# Supplementary material for: Multiple white flat lesions on upper endoscopy: a systematic review and meta-analysis of the association with proton pump inhibitor exposure
Source: BMC Gastroenterol. 2026 May 7;26:392. doi: 10.1186/s12876-026-04771-z (PMC13321529; doi:10.1186/s12876-026-04771-z)
Supplement: Supplementary file 1 — Additional file 1. Supplementary Appendix S1. Full electronic search strategies (last search: November 30, 2025). [file 12876_2026_4771_MOESM1_ESM.pdf]

Supplementary Appendix S1. Full electronic search strategies (last search: November 30, 2025).

Database details	Search strategy (verbatim)
PubMed/MEDLINE Search date: 30 Nov 2025<br>Limits: 2010-01-01 to 2025-11-30; humans (where available); no language restrictions<br>Fields: Title/Abstract (tiab)	(("multiple white flat elevated lesion*" OR "multiple white and flat elevated lesion*" OR "multiple white flat lesion*" OR "white flat elevated mucosa" OR "white flat elevated lesion*" OR "multiple white flat elevated" OR "multiple white flat" OR MWFL OR WFEM OR "whitish flat elevated lesion*")[tiab]) AND ((stomach OR gastric)[tiab]) AND ((endoscop* OR gastroscop* OR gastritis OR "proton pump inhibitor*" OR PPI OR "Kyoto classification")[tiab])
Embase<br>Search date: 30 Nov 2025<br>Limits: 2010-01-01 to 2025-11-30; humans (where available); no language restrictions<br>Fields: Title/Abstract/Keywords (ti,ab,kw)	('multiple white flat elevated lesion*':ti,ab,kw OR 'multiple white and flat elevated lesion*':ti,ab,kw OR 'multiple white flat lesion*':ti,ab,kw OR 'white flat elevated mucosa':ti,ab,kw OR 'white flat elevated lesion*':ti,ab,kw OR mwfl:ti,ab,kw OR wfem:ti,ab,kw OR 'whitish flat elevated lesion*':ti,ab,kw) AND (stomach:ti,ab,kw OR gastric:ti,ab,kw) AND (endoscop*:ti,ab,kw OR gastroscop*:ti,ab,kw OR gastritis:ti,ab,kw OR 'proton pump inhibitor*':ti,ab,kw OR ppi:ti,ab,kw OR 'kyoto classification':ti,ab,kw)
Scopus Search date: 30 Nov 2025 Limits: 2010-01-01 to 2025-11-30; no language restrictions Fields: TITLE-ABS-KEY	TITLE-ABS-KEY("multiple white flat elevated lesion*" OR "multiple white and flat elevated lesion*" OR "multiple white flat lesion*" OR "white flat elevated mucosa" OR "white flat elevated lesion*" OR "whitish flat elevated" OR MWFL OR WFEM) AND TITLE-ABS-KEY(stomach OR gastric) AND TITLE-ABS-KEY(endoscop* OR gastroscop* OR gastritis OR "proton pump inhibitor*" OR PPI OR "Kyoto classification")
Web of Science Core Collection Search date: 30 Nov 2025<br>Limits: Timespan 2010-2025; no language restrictions Fields: Topic (TS)	TS=(<br><br>("multiple white flat elevated"<br><br>OR "multiple white and flat elevated" OR "multiple white flat lesion*" OR "white flat elevated mucosa" OR "white flat elevated lesion*" OR "whitish flat elevated"<br><br>OR "MWFL" OR "WFEM") AND<br><br>(stomach OR gastric) AND<br><br>(endoscop* OR gastroscop*)<br>)
CENTRAL	Search date: 30 Nov 2025<br>Limits: Publication Year from 2010 to 2025; no language restrictions<br>Fields: Title, Abstract, Keywords<br>Search query:<br>("multiple white flat elevated" OR "multiple white and flat elevated" OR MWFL OR WFEM OR "white flat elevated mucosa") AND (gastric OR stomach)
CNKI (中国知网)<br>Search date: 30 Nov 2025<br>Limits: 2010-01-01 to 2025-11-30; 不限语言 Fields: 主题/篇名/关键词	(多发性白色扁平隆起 OR 多发白色扁平隆起 OR 白色扁平隆起 OR 白色扁平隆起性病变)
Wanfang Data (万方) Search date: 30 Nov 2025<br>Limits: 2010-01-01 to 2025-11-30; 不限语言 Fields: 题名/关键词/摘要	("多发白色扁平隆起" OR "白色扁平隆起黏膜" OR "白色扁平隆起 病变" OR "多发性白色扁平隆起" OR MWFL OR WFEM) AND (" 胃" OR "胃体" OR "胃底" OR "胃黏膜") AND ("内镜" OR "胃镜" OR "上消化道内镜")
CQVIP/VIP (维普) Search date: 30 Nov 2025<br>Limits: 2010-01-01 to 2025-11-30; 不限语言 Fields: 题名/关键词/摘要	("多发白色扁平隆起" OR "白色扁平隆起黏膜" OR "白色扁平隆起 病变" OR "多发性白色扁平隆起" OR MWFL OR WFEM) AND (" 胃" OR "胃体" OR "胃底" OR "胃黏膜") AND ("内镜" OR "胃镜" OR "上消化道内镜")
CiNii Articles (Japan) Search date: 30 Nov 2025<br>Limits: 2010-01-01 to 2025-11-30; no language restrictions Fields: All fields	("白色扁平隆起" OR "白色 扁平 隆起" OR "白色扁平隆起粘膜" OR "白色扁平隆起病変" OR "white flat elevated mucosa" OR MWFL OR WFEM) AND (胃 OR gastric OR stomach) AND (内視鏡 OR 胃内視鏡 OR endoscop*)
J-STAGE (Japan)<br>Search date: 30 Nov 2025<br>Limits: 2010-01-01 to 2025-11-30; no language restrictions Fields: All fields	(title:"白色扁平隆起" OR abstracttext:"白色扁平隆起" OR keyword:"白色扁平隆起" OR title:"白色扁平隆起性病変" OR abstracttext:"白色扁平隆起性病変" OR keyword:"白色扁平隆起性 病変" OR title:"白色扁平隆起病変" OR abstracttext:"白色扁平隆起 病変" OR keyword:"白色扁平隆起病変" OR title:"多発性白色扁平 隆起" OR abstracttext:"多発性白色扁平隆起" OR keyword:"多発性 白色扁平隆起" OR title:"春間・川口病変" OR abstracttext:"春間・ 川口病変" OR keyword:"春間・川口病変" OR title:"multiple white and flat elevated" OR abstracttext:"multiple white and flat elevated"<br>OR keyword:"multiple white and flat elevated" OR title:"white flat elevated" OR abstracttext:"white flat elevated" OR keyword:"white flat elevated" OR title:MWFL* OR abstracttext:MWFL* OR keyword:MWFL*)
KoreaMed (Korea) 30 Nov 2025<br><br>Title/Abstract; 2010–2025; no language restriction	("multiple white flat elevated" OR "multiple white and flat elevated" OR "white flat elevated lesion" OR "whitish flat elevated" OR "white flat elevated mucosa" OR MWFL OR WFEM) AND (stomach OR gastric) AND (endoscop* OR gastroscop* OR gastritis OR "proton pump inhibitor*" OR PPI OR "Kyoto classification")

**Database details**	**Search strategy (verbatim)**
RISS (Korea) 30 Nov 2025<br><br>All fields; 2010–2025; no language restriction	("multiple white flat elevated lesions") OR ("MWFL") OR ("White Flat Elevated Mucosa") OR ("WFEM") OR ("위 백색 편평 병변")

The electronic search strategies were jointly developed and implemented by two reviewers (L. Zhang and J. Liu).
