# Supplementary material for: Multiple white flat lesions on upper endoscopy: a systematic review and meta-analysis of the association with proton pump inhibitor exposure
Source: BMC Gastroenterol. 2026 May 7;26:392. doi: 10.1186/s12876-026-04771-z (PMC13321529; doi:10.1186/s12876-026-04771-z)
Supplement: Supplementary file 2 — Additional file 2. Supplementary Tables S1-S8. Supplementary tables (frequencies, exclusions, NOS, ROBINS-I, covariates, descriptive unadjusted proportions). [file 12876_2026_4771_MOESM2_ESM.pdf]

Table S1. Study-reported MWFLs detection frequencies by endoscopy cohort and PPI exposure.

Group	Study	Events/Total	Detection frequency (%)	Notes
Unselected cohort	Adachi et al. 2018 [15]	60/1995	3.0%	Very few PPI users; no clear inference about the PPI association in this cohort.
Unselected cohort	Hatano et al. 2018 [17]	59/1214	4.9%	Current PPI use and prior H. pylori eradication were associated with higher detection frequency.
Unselected cohort	Majima et al. 2018 [16]	80/767	10.4%	Long-term PPI use was reported to be associated with MWFLs; follow-up subset reported.
Unselected cohort	Zhou et al. 2020 [18]	75/926	8.1%	Chinese single-center cohort; pathology described as hyperplastic polyp-like changes.
Enriched cohort	Hasegawa et al. 2024 [5]	35/71 vs 10/92	49.3% vs 10.9%	H. pylori-negative long-term PPI users vs non-users; included for descriptive contrast only (not population-representative).

Note: *H. pylori-negative sample; it is not population-representative and should not be interpreted as population prevalence. Study characteristics and exposure definitions are summarized in Table 1. Frequencies are reported as events/total (%) as extracted from each study. Unselected cohorts represent screening or consecutive routine endoscopy populations not enriched for MWFLs or PPI exposure. The prospective observational cohort is included to illustrate the contrast between long-term PPI users and non-users in an enriched H. pylori-negative cohort.*

Table S2. Reports excluded after eligibility assessment (n = 7) and primary reasons.

Note: *Duplicate reports (including Epub ahead-of-print vs final publication) were handled by retaining the most complete report and consolidating duplicates to avoid double-counting. Duplicate database records were removed during de-duplication and are not counted separately here. Bracketed numbers indicate the corresponding reference number in the main bibliography (where available).*

Excluded report	Primary reason for exclusion
Hasegawa et al. 2018 [33] (Clin Endosc). "Magnified Endoscopic Findings of Multiple White Flat Lesions: A New Subtype of Gastric Hyperplastic Polyps in the Stomach." DOI: 10.5946/ce.2018.104.	Insufficient extractable data: Original descriptive study focused on magnified endoscopic/histologic characterization; exposure was reported as 'acid-reducing drugs' without extractable PPI-specific effect estimates aligned with prespecified analyses.
Zhou & Duan 2023 [34] (Heilongjiang Medical Journal). Clinical characteristics of 51 cases of gastric multiple white flat elevated lesions. DOI: 10.3969/j.issn.1004-5775.2023.21.002.	Insufficient extractable data: Case series of MWFL-positive patients only; no denominator or comparator group for frequency/association analyses.
Suda et al. 2024 [20] (Diagnostics). "Effects of medication period and gastrin levels on endoscopic gastric mucosal changes in long-term proton pump inhibitor users." DOI: 10.3390/diagnostics14222540.	Evaluated other endoscopic gastric mucosal changes among long-term PPI users; MWFL/WFEM outcomes were not reported with extractable data.
Luo et al. 2023 [21] (Front Med). "Correlation analysis of endoscopic manifestations and eradication effect of Helicobacter pylori." DOI: 10.3389/fmed.2023.1259728.	Outcome not MWFL/WFEM (focused on endoscopic manifestations and eradication response of H. pylori).
Uedo 2017 [1] (Endoscopy). "Multiple white flat lesions are neither intestinal metaplasia nor neoplastic lesions." DOI: 10.1055/s-0043-106434.	Letter/editorial; no original cohort data or extractable quantitative outcomes.
Kim GH 2021 [6] (Gut Liver). "Proton Pump Inhibitor-Related Gastric Mucosal Changes." DOI: 10.5009/gnl20036.	Review article; no original MWFL/WFEM frequency or effect estimates.
Shinozaki et al. 2024 [27] (DEN Open). "Endoscopic findings and outcomes of gastric mucosal changes relating to potassium-competitive acid blocker and proton pump inhibitor therapy." DOI: 10.1002/deo2.400.	Original study of broader acid-suppressant-related gastric mucosal changes; no extractable MWFL/WFEM-specific frequency or effect estimates suitable for this review's prespecified analyses.

Table S3. Newcastle–Ottawa Scale (NOS) quality assessment for included studies.

Study	Selection (0–4)	Comparability (0–2)	Outcome (0–3)	Total
Adachi et al. 2018 [15]	3	2	2	7
Hatano et al. 2018 [17]	3	2	2	7
Majima et al. 2018 [16]	4	2	2	8
Hasegawa et al. 2024 [5]	3	1	2	6
Zhou et al. 2020 [18]	3	1	2	6

Table S4. ROBINS-I risk of bias judgments by domain for included studies.

Study	Confounding	Selection	Classification	Deviations	Missing data	Measurement	Reporting	Overall	Key concerns/notes
Adachi et al. (2018) [15]	Moderate	Low	Moderate	Low	Low	Moderate	Low	Moderate	Limited adjustment; possible detection bias; exposure duration may be imprecise.
Hatano et al. (2018) [17]	Moderate	Low	Moderate	Low	Low	Low	Low	Moderate	Residual confounding; exposure defined as current use; potential detection bias.
Majima et al. (2018) [16]	Moderate	Low	Low	Low	Low	Low	Low	Moderate	Residual confounding; subset follow-up data; exposure definition clearer.
Hasegawa et al. (2024) [5]	Moderate	Moderate	Low	Low	Low	Low	Low	Moderate	Selection/enrichment (selected cohort); residual confounding by indication; limited generalizability.
Zhou et al. (2020) [18]	Moderate	Low	Low	Low	Low	Low	Low	Moderate	Limited adjustment; exposure definition may be heterogeneous; outcome definition partly pathology-based.

Table S5. Study characteristics: country, design, population, exposure definition, and key findings.

Study	Country	Design/Setting	Population	Exposure definition	Key findings
Adachi et al. 2018 [15]	Japan	Cross-sectional; single-center screening endoscopy	Health check-up EGD; N=1,995	Current PPI use (very few users)	More frequent after H. pylori eradication; inference about the PPI association was limited by very few users.
Hatano et al. 2018 [17]	Japan	Cross-sectional; multicenter routine diagnostic endoscopy	Outpatients undergoing EGD; N=1,214	Current PPI use vs non-use	PPI use and longer exposure duration were associated with higher MWFL detection; markedly lower detection was reported with active H. pylori infection.
Majima et al. 2018 [16]	Japan	Prospective study; screening endoscopy with follow-up subset	Screening EGD; N=767	Long-term PPI use (≥6 months)	Long-term PPI use was reported to be associated with MWFLs; lesions were largely stable during limited follow-up, and no malignant transformation was reported.
Hasegawa et al. 2024 [5]	Japan	Prospective observational; single-center	H. pylori-negative patients: 71 long-term PPI users vs 92 non-users (N=163)	Oral PPI intake (long-term)	MWFL frequency was approximately 4.5-fold higher among PPI users; PPI intake remained independently associated with MWFLs in this enriched cohort.
Zhou et al. 2020 [18]	China	Cross-sectional; single-center diagnostic endoscopy	Consecutive diagnostic EGD; N=926	Current PPI use vs non-use	MWFLs were more common among PPI users; pathology was described as hyperplastic polyp-like changes.

Table S6. Compilation of exposure definitions, covariates, and adjusted effect estimates used for PPI–MWFLs association analyses.

These data were used for the PPI–MWFLs association analyses, exactly as reported in the original studies.

Study (design)	Exposure definition and adjusted model covariates (as reported)	Effect estimate used in this review
Hatano et al. 2018 [17] (cross-sectional)	Exposure definition: current PPI use vs non-use (as recorded at endoscopy). Covariates: Age; sex; BMI; comorbidities (renal dysfunction, liver cirrhosis, hypertension, diabetes mellitus); H. pylori eradication; atrophic gastritis; medications (PPI, H2 blocker, corticosteroids).	Primary adjusted OR meta-analysis: 3.58 (95% CI 1.94–6.61).
Majima et al. 2018 [16] (prospective study; baseline estimate analyzed cross-sectionally)	Exposure definition: long-term PPI use (≥6 months) vs non-use. Covariates: Age; sex; H. pylori infection status (no infection / current infection / previous infection).	Primary adjusted OR meta-analysis: 3.51 (95% CI 1.92–6.42).
Zhou et al. 2020 [18] (cross-sectional)	Exposure definition: current PPI use vs non-use (duration threshold not specified). Covariates: Age (≥55 years); sex.	Primary adjusted OR meta-analysis: 1.95 (95% CI 1.05–3.62).
Hasegawa et al. 2024 [5] (prospective observational)	Exposure definition: long-term oral PPI intake vs non-use in an enriched H. pylori-negative cohort. Covariates: Age; sex; hypergastrinemia; H. pylori infection status; fundic gland polyps; foveolar-hyperplastic polyps; atrophy of surrounding mucosa.	Sensitivity analysis (including prospective cohort): 5.78 (95% CI 2.06–16.21).

Table S7. Study contribution matrix for each quantitative synthesis.

Study	Adjusted OR meta-analysis (Fig 3)	Adjusted OR + prospective (Fig S5)	Crude OR sensitivity (Fig 4)	H. pylori status (Fig S2)	Follow-up data
Adachi et al. 2018 [15]			✓		
Hatano et al. 2018 [17]	✓	✓	✓	✓	
Majima et al. 2018 [16]	✓	✓	✓	✓	✓
Hasegawa et al. 2024 [5]		✓		✓	
Zhou et al. 2020 [18]	✓	✓	✓		

Table S8. Descriptive unadjusted MWFL proportions by PPI exposure status.

Study	PPI users (MWFLs/total, %)	Non-PPI users (MWFLs/total, %)
Hatano 2018 [17]	25/188 (13.3%)	34/1026 (3.3%)
Majima 2018 [16]	20/76 (26.3%)	58/669 (8.7%)
Zhou 2020 [18]	53/264 (20.1%)	22/662 (3.3%)
**Sum excluding Hasegawa 2024 [5] (3 studies; descriptive)**	**98/528 (18.6%)**	**114/2357 (4.8%)**
Hasegawa 2024 [5] (prospective observational)	35/71 (49.3%)	10/92 (10.9%)
**Sum of extracted counts (4 studies; descriptive)**	**133/599 (22.2%)**	**124/2449 (5.1%)**

Note: *Numbers shown are participants with MWFLs/total participants (%). Event proportions are purely descriptive and unadjusted. One prospective observational study (Hasegawa 2024 [5]) is included in the summary row; these event proportions are not representative of the general population and should not be read as population prevalence estimates. For reference, we provide a separate summary that excludes Hasegawa 2024 [5]. Denominators may not match study totals since some studies did not report PPI exposure data for everyone. The Adachi et al. (2018) study reported too few PPI users to be included in Table S8, although we did include it in the crude OR sensitivity analysis (Figure 4).*
