# Supplementary material for: Multiple white flat lesions on upper endoscopy: a systematic review and meta-analysis of the association with proton pump inhibitor exposure
Source: BMC Gastroenterol. 2026 May 7;26:392. doi: 10.1186/s12876-026-04771-z (PMC13321529; doi:10.1186/s12876-026-04771-z)

Figure S1. Study-reported MWFLs detection frequency in unselected endoscopy cohorts.

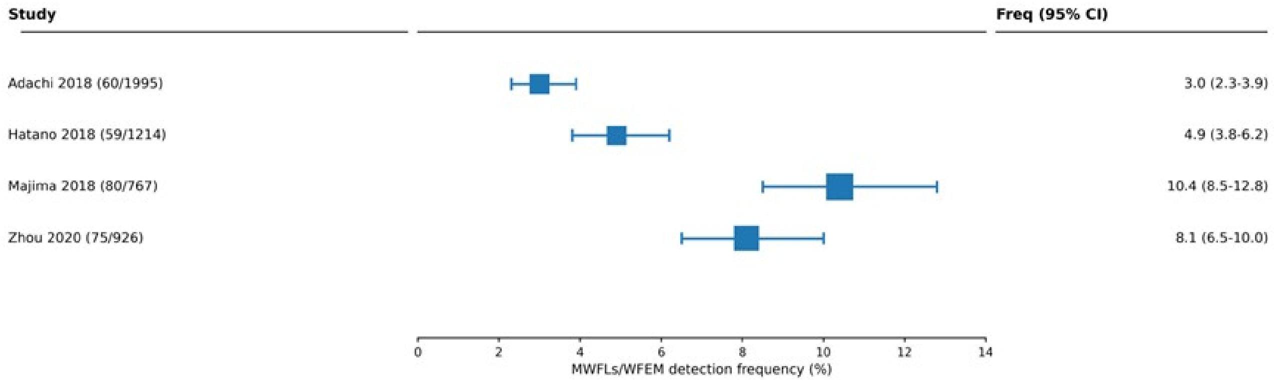

Figure S2. MWFL occurrence by *H. pylori* status. The comparison contrasts *H. pylori*-negative (never infected or post-eradication) and *H. pylori*-positive groups in two cross-sectional studies from Japan. Modeled via random-effects REML, this comparison is exploratory, reflecting both the limited study count and varying *H. pylori* definitions.

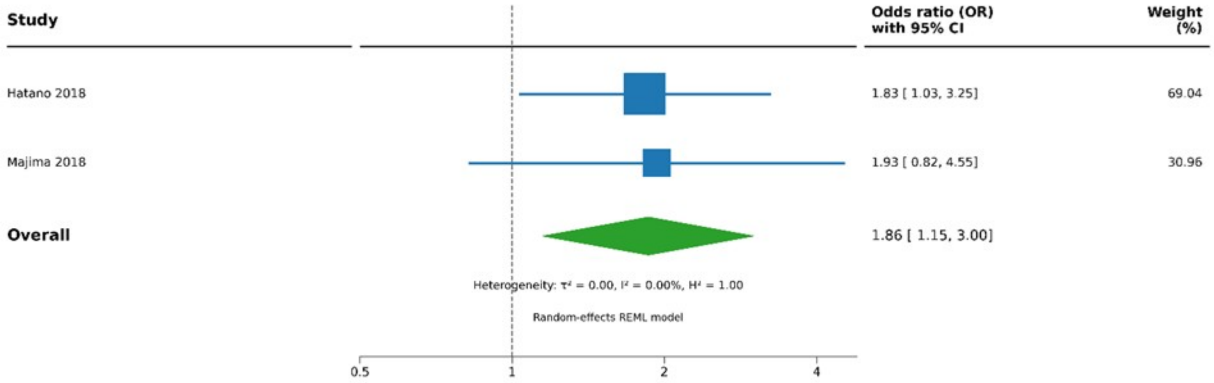

Figure S3. Quality of included studies assessed by the Newcastle–Ottawa Scale (NOS).

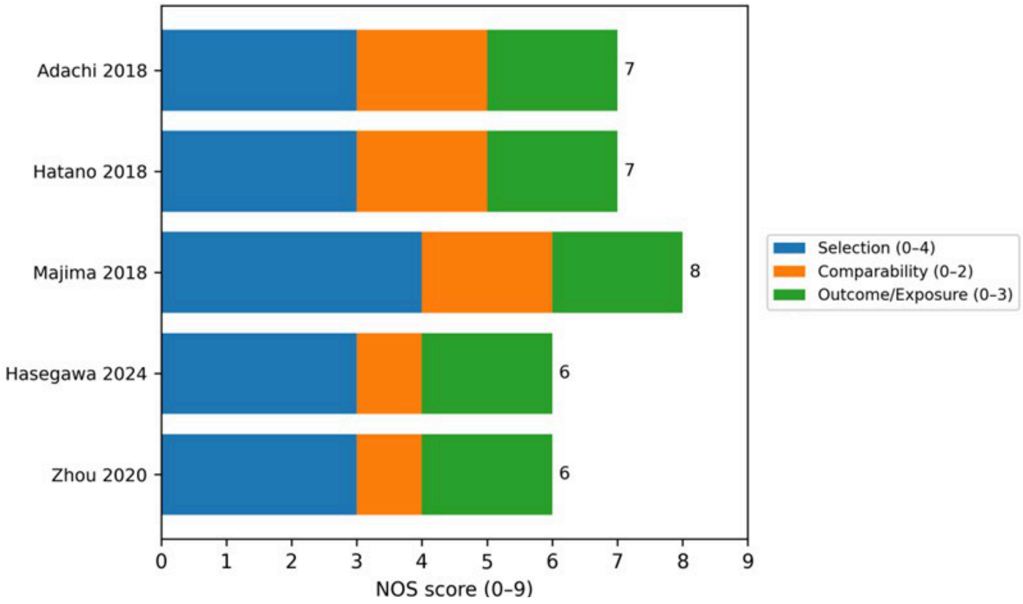

Figure S4. Sensitivity analysis including one prospective observational study. A random-effects REML model was used for this forest plot to show the PPI-MWFL association when one prospective study was included alongside the three cross-sectional studies (k=4; Japan n=3, China n=1). This synthesis is observational and serves an exploratory purpose.

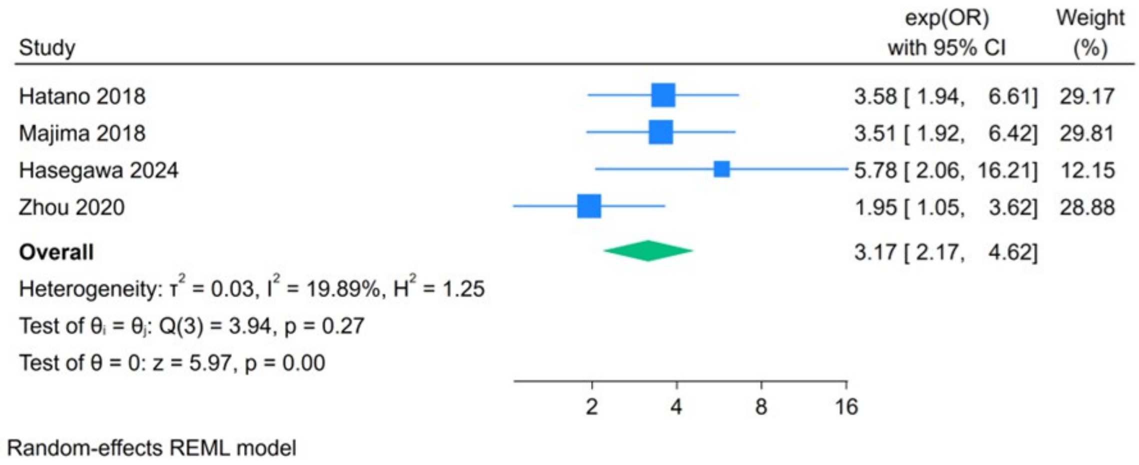

Figure S5. Funnel plot depicting the crude odds ratio sensitivity analysis (k=5).
Due to the small number of studies, interpretation is limited; the plot is thus provided for descriptive purposes only.

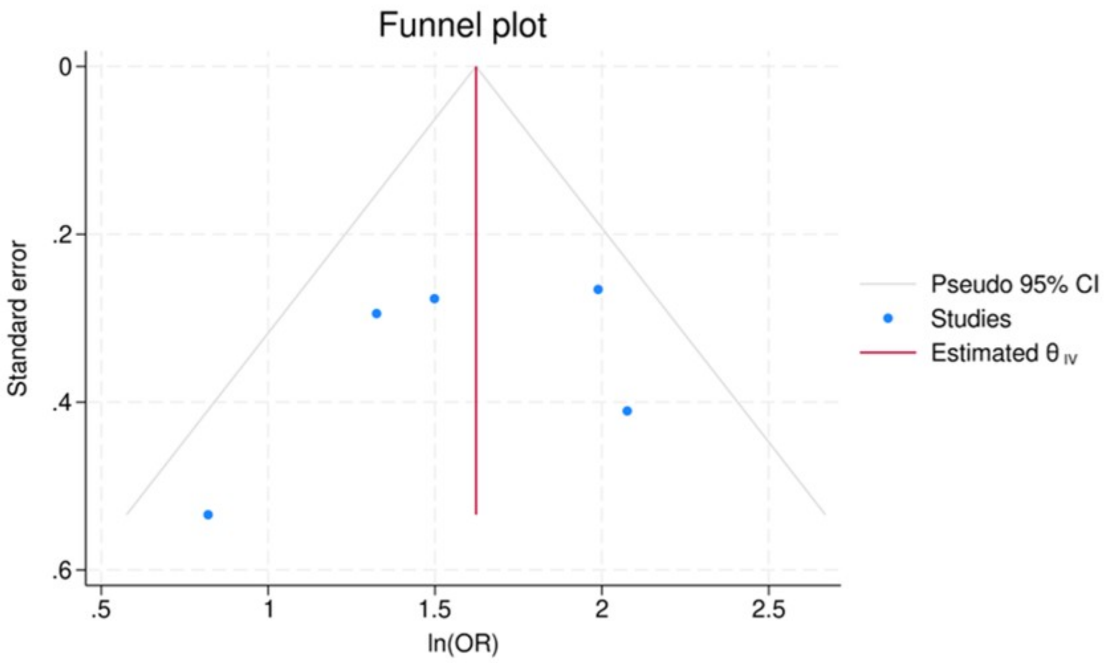

Figure S6. Illustrative endoscopic features of gastric MWFLs/WFEM. Panels A (WLI) and B (NBI) correspond to the fundus; Panels C (WLI) and D (NBI), to the body. These anonymized images serve a purely illustrative function and were not extracted as review data.

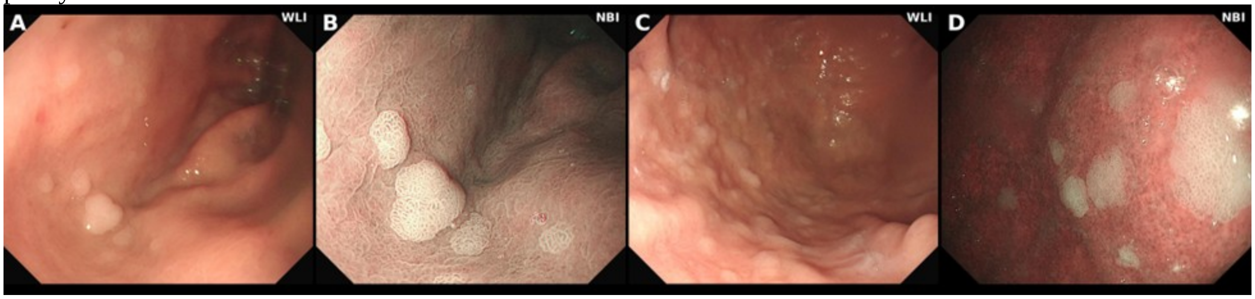

Supplement: Supplementary file 3 — Additional file 3. Supplementary Figures S1–S6. Supplementary figures (frequency plot, forest plots, quality and risk-of-bias summaries, funnel plot, illustrative endoscopic images). [file 12876_2026_4771_MOESM3_ESM.pdf]
