# Supplementary material for: Multiple white flat lesions on upper endoscopy: a systematic review and meta-analysis of the association with proton pump inhibitor exposure
Source: BMC Gastroenterol. 2026 May 7;26:392. doi: 10.1186/s12876-026-04771-z (PMC13321529; doi:10.1186/s12876-026-04771-z)
Supplement: Supplementary file 4 — Additional file 4. PRISMA 2020 checklist. [file 12876_2026_4771_MOESM4_ESM.docx]

**PRISMA 2020 Checklist - Detailed Formal Version**

Manuscript: Multiple white flat lesions on upper endoscopy: a systematic review and meta-analysis of the association with proton pump inhibitor exposure

|  |
| --- |

| This submission-ready version expands the original location matrix into fuller reporting statements. Obvious supplementary figure cross-reference mismatches visible in the uploaded materials were standardized for clarity in this formal version (for example, the descriptive funnel plot is cited as Figure S5). |
| --- |

Location references use manuscript sections, tables, figures, and additional files.

# **Title and Abstract**

| **Item** | **PRISMA 2020 checklist item** | **Detailed reporting statement** | **Location where reported** |
| --- | --- | --- | --- |
| 1 | Identify the report as a systematic review and meta-analysis. | The title page identifies the manuscript as a systematic review and meta-analysis examining multiple white flat lesions on upper endoscopy and their association with proton pump inhibitor exposure. | Title page |
| 2 | Provide a structured abstract summarizing background, methods, results, limitations, and conclusions. | The abstract is structured and summarizes the clinical background, search and eligibility methods, included evidence, principal findings, key limitations, and the overall conclusion. | Abstract |

# **Introduction**

| **Item** | **PRISMA 2020 checklist item** | **Detailed reporting statement** | **Location where reported** |
| --- | --- | --- | --- |
| 3 | Describe the rationale for the review in the context of existing knowledge. | The Background section explains the clinical relevance of MWFLs/WFEM and sets out why a systematic synthesis of the possible relationship with PPI exposure is needed. | Background |
| 4 | State the review question/objectives, including key components of eligibility. | The final paragraph of the Background states the review objective and frames the review question around upper-endoscopic MWFLs/WFEM, eligibility, and the association with PPI exposure. | Background (final paragraph) |

# **Methods**

| **Item** | **PRISMA 2020 checklist item** | **Detailed reporting statement** | **Location where reported** |
| --- | --- | --- | --- |
| 5 | Specify inclusion and exclusion criteria and how studies were grouped. | Methods: Eligibility criteria defines which studies, populations, outcomes, and exposures were eligible and explains how studies were grouped for descriptive and quantitative syntheses. | Methods: Eligibility criteria |
| 6 | List all information sources and the date of the last search. | Methods: Data sources and search strategy lists all databases and information sources and specifies the date of the last search. | Methods: Data sources and search strategy |
| 7 | Present the full search strategies for all databases and sources. | Full database-specific search strategies are reported in Supplementary Appendix S1 so the electronic search can be reproduced. | Additional file 1 (Supplementary Appendix S1) |
| 8 | Describe how records were screened and studies selected. | Methods: Selection process describes the screening workflow and study-selection process, and Figure 1 presents the record flow diagram. | Methods: Selection process; Figure 1 |
| 9 | Describe how data were extracted and checked. | Methods: Data extraction explains how study data were extracted, checked, and prepared for synthesis. | Methods: Data extraction |
| 10 | Define all outcomes and other variables sought. | Outcomes, exposure variables, covariates, and other extracted data items are defined across Methods: Data extraction, Table 1, and Supplementary Tables S5-S7. | Methods: Data extraction; Table 1; Additional file 2 (Tables S5-S7) |
| 11 | Describe the methods used to assess risk of bias in included studies. | Methods: Study quality assessment specifies the risk-of-bias approach, with supporting reporting in Table 3 and Supplementary Tables S3-S4. | Methods: Study quality assessment; Table 3; Additional file 2 (Tables S3-S4) |
| 12 | Specify the effect measures used for each synthesis. | Methods: Data synthesis states the effect measures used for each synthesis, including odds ratios and study-level frequencies where appropriate. | Methods: Data synthesis |
| 13 | Describe synthesis methods, heterogeneity assessment, and sensitivity analyses. | Methods: Data synthesis describes the meta-analytic approach, heterogeneity assessment, and sensitivity analyses undertaken. | Methods: Data synthesis |

# **Results**

| **Item** | **PRISMA 2020 checklist item** | **Detailed reporting statement** | **Location where reported** |
| --- | --- | --- | --- |
| 14 | Report the results of the search and selection process. | Results: Study selection and Figure 1 report records identified, screened, excluded, and included; excluded full-text reports and reasons are itemized in Supplementary Table S2. | Results: Study selection; Figure 1; Additional file 2 (Table S2) |
| 15 | Present characteristics of included studies. | Study characteristics are summarized in Table 1 and Results: Study characteristics, with additional design, exposure, and synthesis-role detail in Supplementary Tables S5-S7. | Table 1; Results: Study characteristics; Additional file 2 (Tables S5-S7) |
| 16 | Present assessments of risk of bias for included studies. | Risk-of-bias assessments are presented in Table 3, Supplementary Tables S3-S4, and Supplementary Figure S3. | Table 3; Additional file 2 (Tables S3-S4); Additional file 3 (Figure S3) |
| 17 | For each synthesis, report study-level results and pooled estimates where applicable. | Study-level and pooled or descriptive synthesis results are reported in the Results section, Figures 2-4, Table 2, and relevant supplementary displays, including Supplementary Figures S1, S2, and S4 and Supplementary Table S7. | Results; Figures 2-4; Table 2; Additional file 3 (Figures S1, S2, and S4); Additional file 2 (Table S7) |
| 18 | Present results of reporting-bias assessment. | Because fewer than 10 studies were available, no formal small-study-effects test was performed; this limitation is stated in Methods/Results, and a funnel plot is provided descriptively as Supplementary Figure S5. | Methods and Results: fewer than 10 studies available, so no formal small-study-effects testing; Additional file 3 (Figure S5, descriptive only) |
| 19 | Present certainty of evidence for each important outcome. | The certainty/strength of evidence for key outcomes is summarized in Table 4 and interpreted further in the Discussion. | Table 4; Discussion |

# **Discussion**

| **Item** | **PRISMA 2020 checklist item** | **Detailed reporting statement** | **Location where reported** |
| --- | --- | --- | --- |
| 20 | Interpret the results in light of the evidence base and other evidence. | The Discussion interprets the findings in the context of the included observational evidence and prior literature on acid-suppressant-related gastric mucosal changes. | Discussion |
| 21 | Discuss limitations of the included evidence. | The Discussion includes a limitations paragraph addressing weaknesses of the included studies and the underlying evidence base. | Discussion; Limitations paragraph |
| 22 | Discuss limitations of the review processes used. | The same limitations paragraph also addresses constraints of the review process itself, including the limited number of studies and observational design of the available evidence. | Discussion; Limitations paragraph |
| 23 | Discuss implications for practice, policy, and future research. | The Conclusions and Discussion identify implications for clinical interpretation and future research; Table 4 also informs the overall evidence interpretation. | Conclusions; Table 4; Discussion |

# **Other Information**

| **Item** | **PRISMA 2020 checklist item** | **Detailed reporting statement** | **Location where reported** |
| --- | --- | --- | --- |
| 24 | Provide registration information and where the protocol can be accessed. | Registration information is reported in the last line of the abstract, and protocol/reporting details are described in Methods: Protocol and reporting. | Abstract (last line); Methods: Protocol and reporting |
| 25 | Describe sources of support and the role of funders. | Sources of support and funding are disclosed in the Declarations: Funding section. | Declarations: Funding |
| 26 | Declare competing interests. | Potential competing interests are disclosed in the Declarations: Competing interests section. | Declarations: Competing interests |
| 27 | Report where data, code, and other materials are available. | Availability of data, code, and related supplementary materials is stated in Declarations: Availability of data and materials and the Additional files section. | Declarations: Availability of data and materials; Additional files section |
